# First case report of horseshoe appendix in Morocco according according to SCARE guidelines

**DOI:** 10.1016/j.amsu.2021.102870

**Published:** 2021-09-20

**Authors:** Rachid Jabi, Siham Elmir, Mohammed Bouziane

**Affiliations:** a Department of General Surgery, Mohammed VI University Hospital, Faculty of Medicine and Pharmacy, Oujda, Morocco; b Laboratory of Anatomy, Microsurgery and Surgery Experimental and Medical Simulation (LAMCESM), Mohammed Ist University, Oujda, Morocco; c Department of Physical Medicine and Rehabilitation, Mohammed VI University Hospital, Faculty of Medicine and Pharmacy, Oujda, Morocco

**Keywords:** Acute appendicitis, Duplication, Horseshoe appendix, Morocco

## Abstract

**Introduction:**

The appendix duplication is a sporadic malformation in which the horseshoe form is the uncommon described variant. To our knowledge, we report the first Moroccan case of a horseshoe appendix in a girl admitted to managing of pain at the right iliac fossa.

**Case presentation:**

Through this article, we present a very rare case of appendicular duplication. It has not been objectified in radiological exploration and discovered by chance during the operation. Resection then closure of the appendicular bases allowed our patient to heal**.** The objectives of this work is threefold: i) to report this sporadic case of horeshoe appendix, ii) to emphasize the importance of suspicion of appendicular duplication in appendicular syndrome and iii) to recommend the exploration of the ileoceacal region to avoid surgical complications and medicolegal problems**.**

**Conclusion:**

Our case report shows that we have to take into consideration this sporadic presentation of appendicular syndrome and this even in the absence of radiological signs. Our work brings enriched the literature by a new case of horseshoe appendicitis highlighting the importance of surgical treatment**.**

## Highlights

• We report a case report of an appendix duplication witch is a very rare congenital malformation. To date, only fifteen cases were published.
• After careful examination of the literature, we found that this is the first case of this rare entity in Morocco.
• Appendicular duplication is a rare malformation that often escapes preoperative radiological imaging.
• We believe that this manuscript can add new perspectives to the surgery practice of this very rare presentation.

## 1 Introduction

Duplication of the vermiform appendix is a sporadic malformation [1]. It is associated with other appendix malformations described in the literature such as a congenital absence of the vermiform appendix and the intracecal appendix [2,3]. The horseshoe appendix is the rarest and the most recently described type of this entity [4]. Several classifications are available to guide surgeons to diagnose this variation [5,6]. Radical surgery is the treatment of choice, enables a successful management, and avoids recurrence, as well as medico-legal problems [[7], [8], [9]]. To date, only few cases describing this anatomical variation were published. We report in this paper the first Moroccan case of a horseshoe appendix according to SCARE guidelines [10] (see Table 1).

**Table 1:** Summary of published case reports on horseshoe appendix.

Author/Year	Country	Diagnosis	Anatomical location	Surgical procedure
Mesko et al., 1989 [11]	USA	Appendicitis	Unclear	Appendectomy
Dong et al., 1994 [12]	China	Bowel occlusion	Cecum-cecum	Appendectomy
Dasgupta et al., 1999 [13]	England	Appendicular mass	Cecum-cecum	Appendectomy
Li and Yu. 2000 [14]	China	Appendicitis	Cecum-cecum	Appendectomy
Cai and Yu. 2006 [15]	China	Appendicitis	Cecum-cecum	Appendectomy + enterotomy
Calota et al., 2010 [16]	Romania	Bowel occlusion	Cecum-cecum	Appendectomy
Ninos et al., 2010 [17]	Greece	B Cell non Hodgkin's lymphoma	Cecum-cecum	Appendectomy + chemotherapy
Dube et al., 2011 [18]	South Africa	Appendicitis	Cecum-hepatic flexure of colon	Appendectomy
Li and Liu. 2012 [19]	China	Appendicitis and bowel occlusion	Cecum-cecum	Appendectomy + enterotomy
Oruç et al., 2013 [20]	Turkey	Appendicitis	Cecum-cecum	Appendectomy
Bulut et al., 2016 [21]	Turkey	Appendicitis	Cecum-cecum	Appendectomy
Singh et al., 2016 [22]	India	Appendicitis	Cecum-cecum	Appendectomy
Takabatake et al., 2016 [23]	Japan	Tubulovillous adenoma in ascending colon	Cecum-ascending colon	Ileoceacal resection
Liu et al., 2017 [4]	China	Appendicular mass	Cecum-cecum	Appendectomy
Zhu et al., 2019 [24]	China	acute appendicitis and endometriosis	Cecum-cecum	Appendectomy, oophorectomy and partial resection of the small intestine
Our case. 2021	Morocco	appendicitis	Cecum-cecum	Appendectomy

## 2 Clinical case

**Fig. 1:**
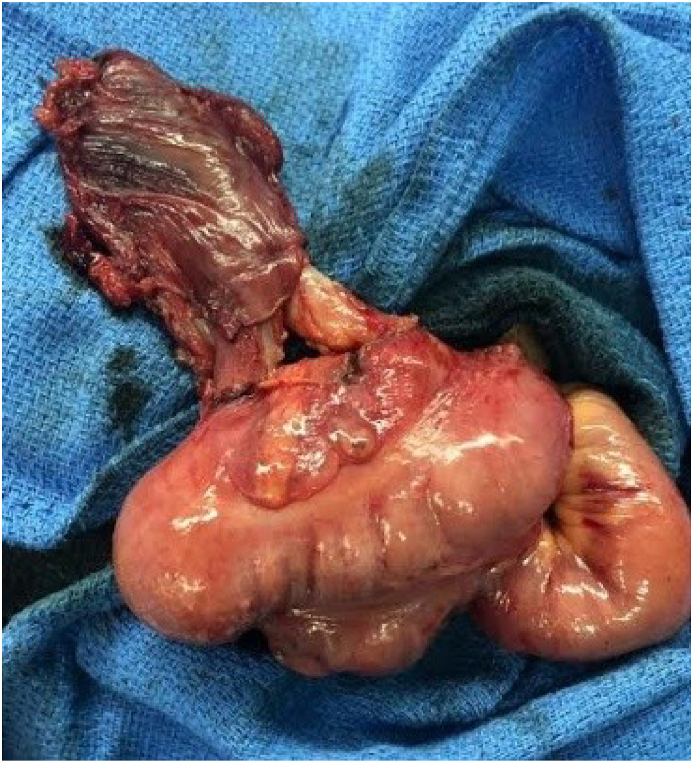
Horsesho appendix with two different bases

A 26-year-old married woman without children from eastern Morocco and under insulin treatment for type I diabetes for 6 years was admitted to the emergency department for ketoacidosis and presented with acute onset of abdominal pain and vomiting. The clinical examination found a patient with fever at 39 °C with a positive urine test strip and tenderness in the right iliac fossa (Alvarado score> 6). We performed an infectious assessment that showed hyperleukocytosis (16,000/mm3), an increase of C-reactive protein (CRP) at 66 mg/L with slight metabolic acidosis on arterial gasometry. Ultrasound examination was in favor of acute appendicitis. After discussion with the patient, the consent for surgical exploration was obtained and showed a horseshoe appendix (Fig. 1). An appendectomy with control of both appendicular bases was performed by the head of the digestive surgery department who was called upon in the operating room by his team in the face of this anomaly. The intervention went well, tolerated by the patient, without any unwanted events. The patient was discharged three days later after the control of ketoacidosis and penicillin-based oral therapy (amoxicillin 3g/day) for 6 days was prescribed. The histopathological examination of the surgical specimen was in favor of suppurative appendicitis and our patient was satisfied with the medical and surgical management.

## 3 Discussion

Duplication of the vermiform appendix is an extremely rare malformation with an estimated

incidence of 0.004% [1]. It was first described by Picoli in 1892 [25]. It also described other

rare anatomical variations such as acute appendicitis with absence of appendix [2] and

appendicular ectopia [3]. Cave classified appendix duplication in 1936 according to the

anatomical location [5]. Since then, this classification was updated by Wallbridge in 1963 [6]

and was named “Cave-Wallbridge”. In 2010, Calotă et al. added the horseshoe shape to the

old classification [4]. Recently other variations such as the triple appendix [26] have been

described. We reported a new case of a horseshoe appendix that is a very rare

morphological entity in addition to the fifteen reported cases described in the literature.

Several hypotheses have explored the genesis of the a horseshoe appendix such as fusion of the two appendicular points, appendiculo-cecal fistula and division of the appendicular mesoappendix explained by the presence of a single vessel in the mesoappendix [22]. The intraoperative fortuitous discovery often dominates diagnosis circumstances for suspicion of appendicitis or during surgery for other indications [[27], [28]]. In our patient, appendicitis was strongly suspected with an Alvarado score greater than 6. Preoperative radiology rarely helps in the diagnosis of appendicular duplication. However, one reported case of fortuitous discovery on a barium radiological examination was described in a patient admitted for other medical conditions [29]. Liu et al. [4] reported two cases of a horseshoe appendix diagnosed preoperatively on ultrasound examination with 3D reconstruction in addition to other cases after re-reading the CT scan after postoperative discovery of this very rare variation during surgery. In our case, the radiology team suspected acute appendicitis, and our surgical exploration found horseshoe appendicitis with a single vessel on the mesoappendix. The evaluation of the cecum must be rigorously performed to avoid any forensic problems if a new inflammation affects the remaining annex [7,8]. It also allows better assessment of differential diagnosis of duplication with the cecal diverticulitis as well as colon tumors [[30], [31]]. Removal of both appendices is important to avoid confusion and additional re-interventions [9]. Moreover, an additional dissection of the cecum should be performed to eliminate duplication at the retrocecal area [16].

## 4 Conclusion

Appendicular duplication is a sporadic malformation that often escapes preoperative radiological imaging. Surgeons must rigorously examine the ileocecal region in order to avoid missing any appendicular duplication in case of symptomatic recurrence, and we insist on the radical surgical treatment which alone allows the cure.

## Patient percepctive

The procedure of surgery was explained to the patient with all advantages and possible complications. He agreed on the procedure and informed consent was taken from her.

## Funding

The author(s) received no financial support for the research, authorship and/or publication of this article.

## Ethics approval

not applicable.

## Consent of patient

Written informed consent was obtained from the patient for publication of this case report and accompanying images. A copy of the written consent is available for review by the Editor-in-Chief of this journal on request.

## Author's contribution

Jabi Rachid: Writing, review and editing of the manuscript.

Siham Elmir: Contributed for diagnose and treatment of the patient.

Mohamed Bouziane: Review, Supervision and surgeons of the patient.

## Registration of research studies

1 Name of the registry:
2 Unique identifying number or registration ID:
3 Hyperlink to your specific registration (must be publicly accessible and will be checked):

## Guarantor

Jabi Rachid.

## Provenance and peer review

Not commissioned, externally peer-reviewed.

## Declaration of competing interest

The authors declared no potential conflicts of interests with respect to research, authorship and/or publication of the article.
